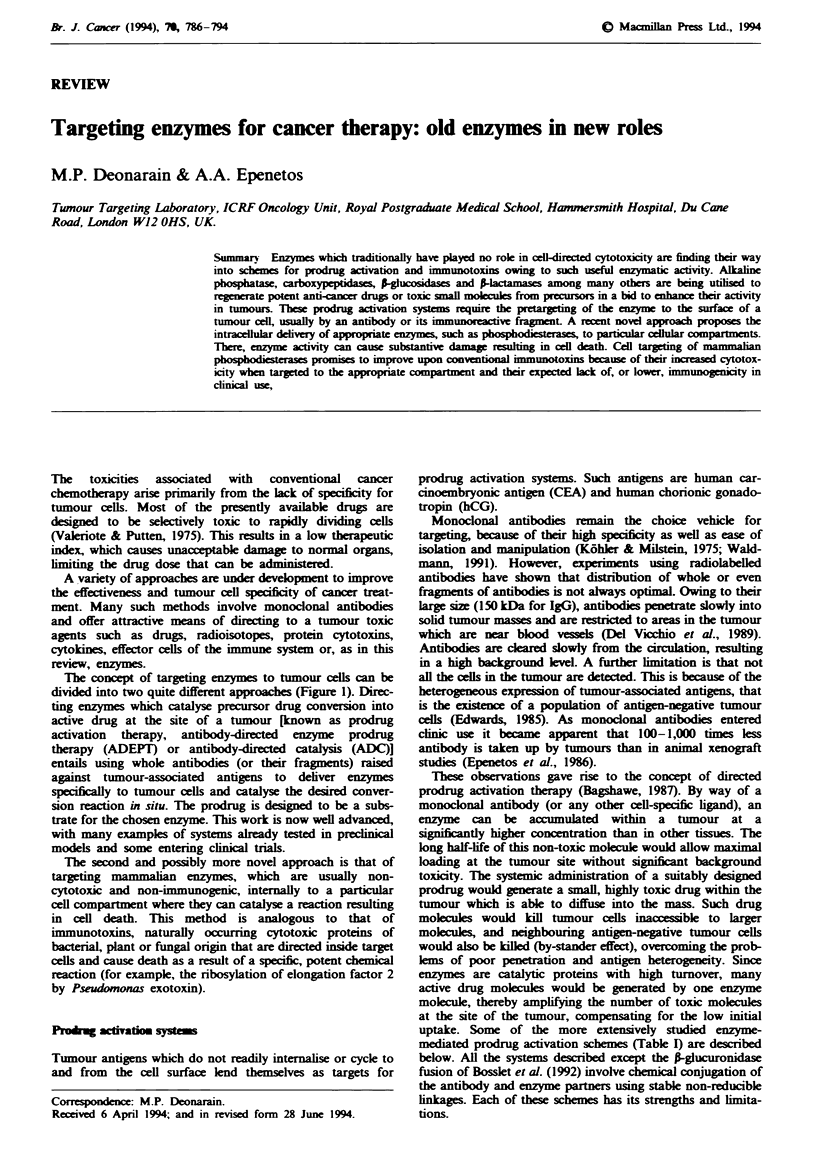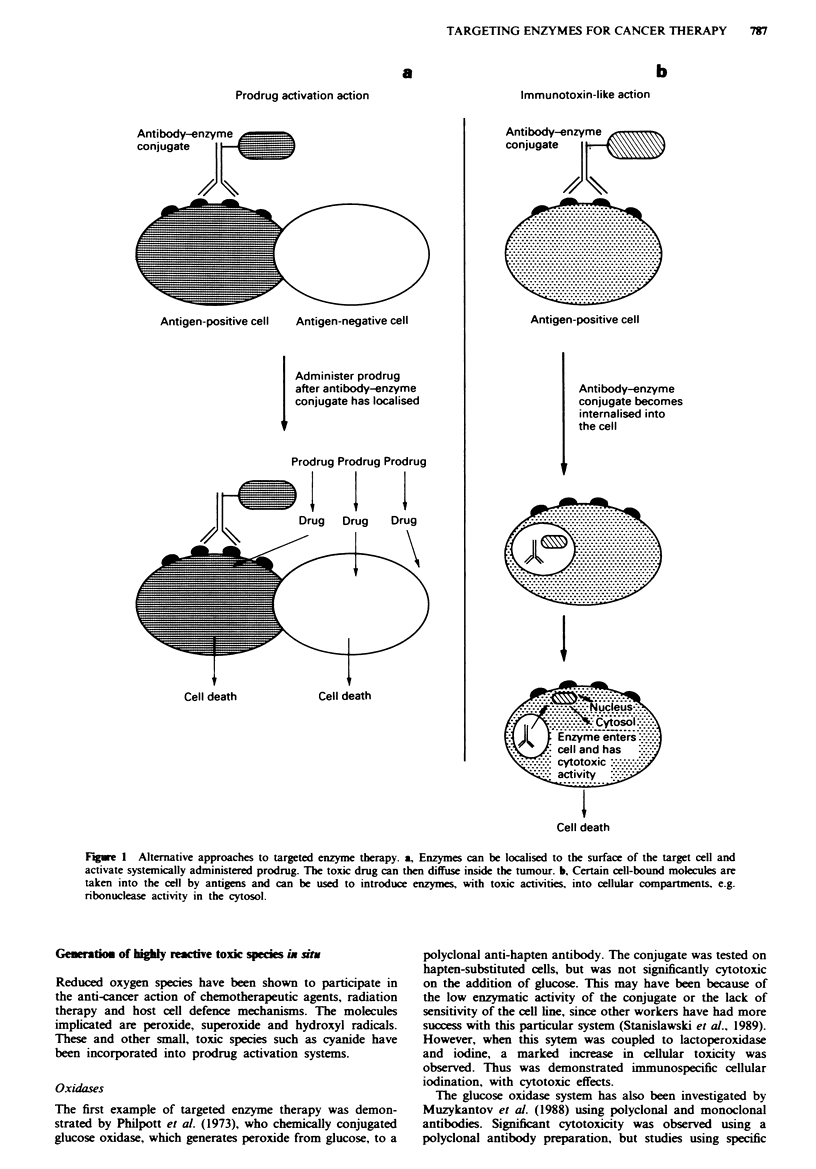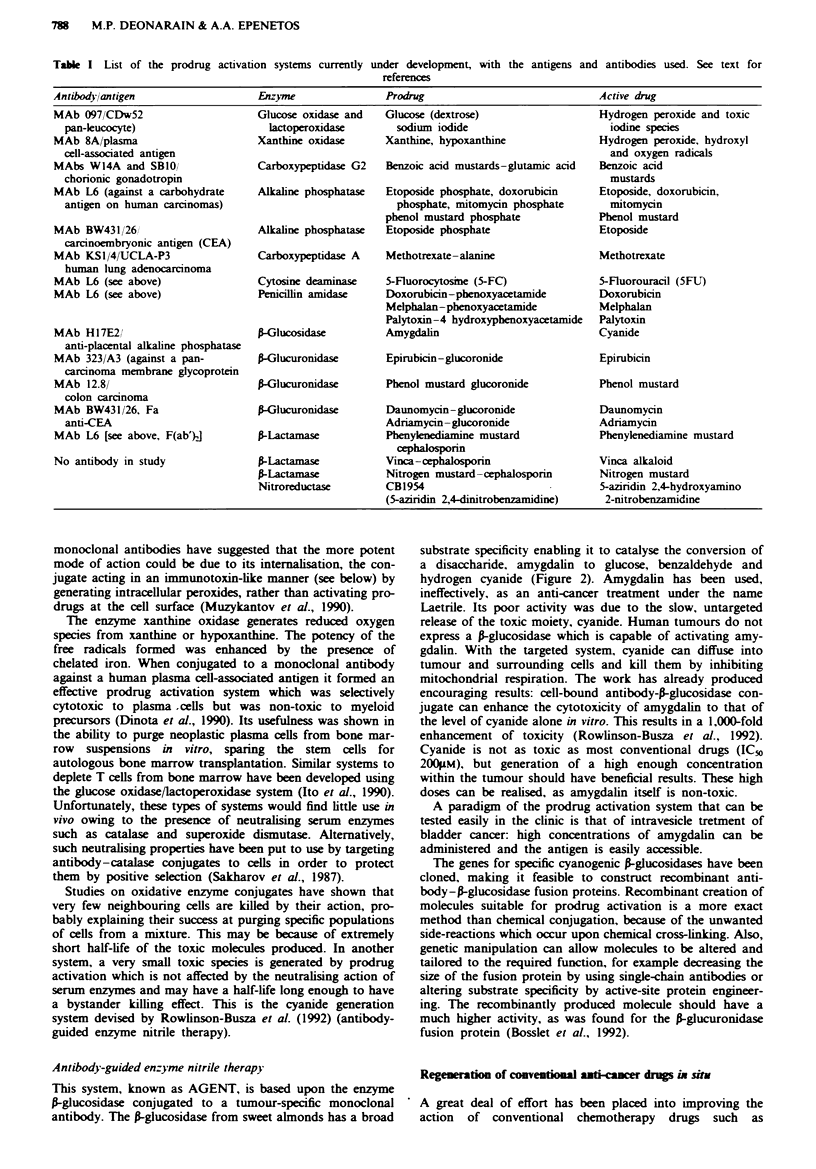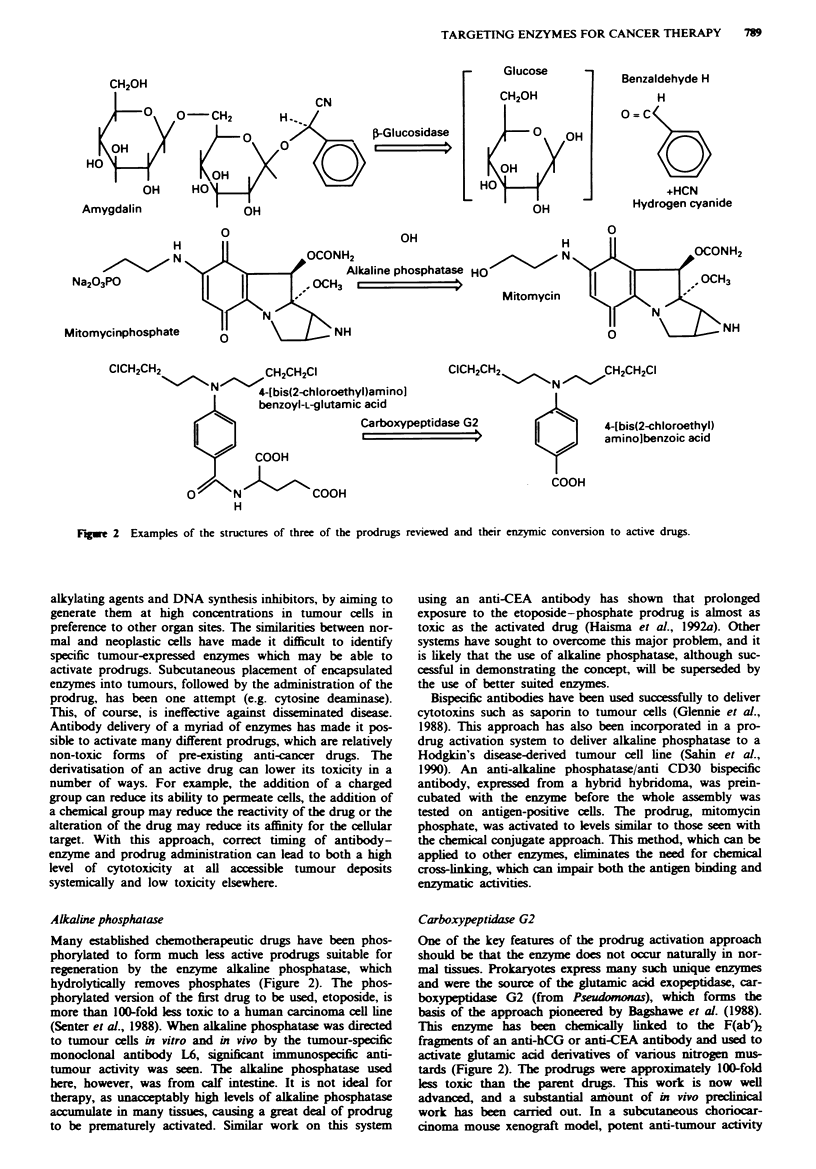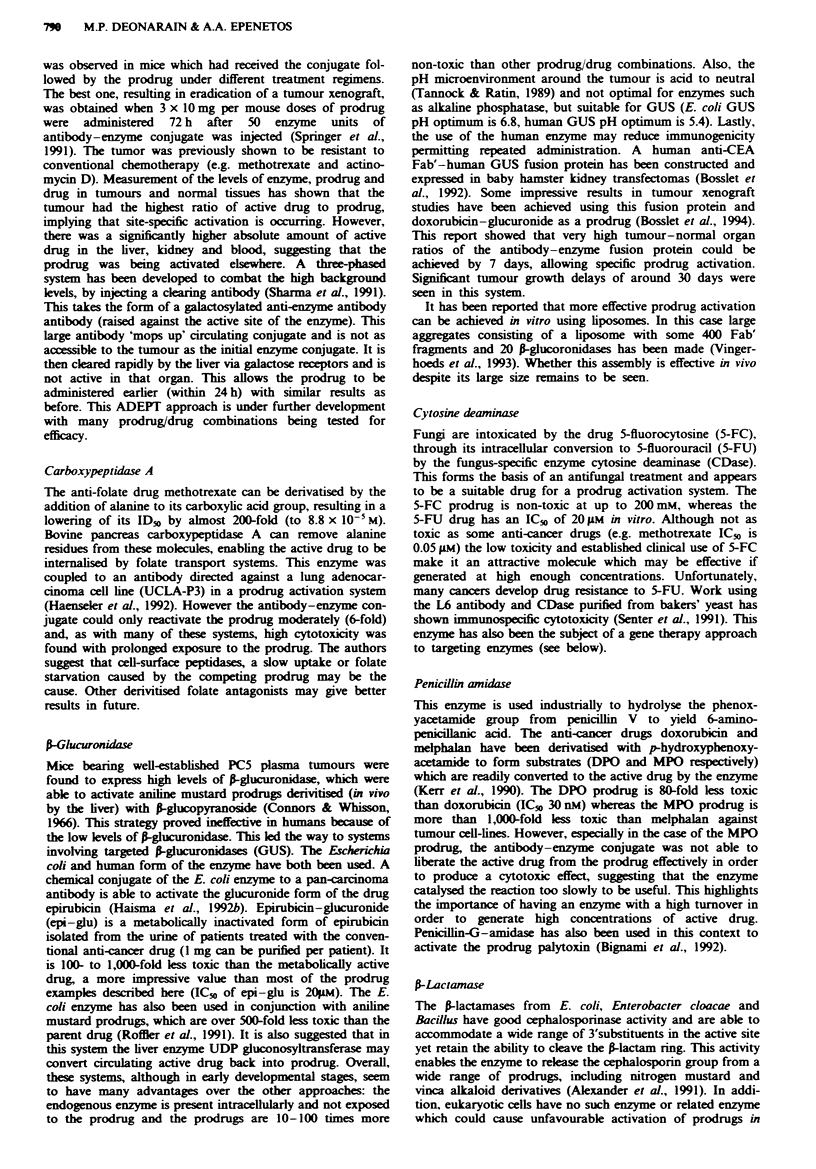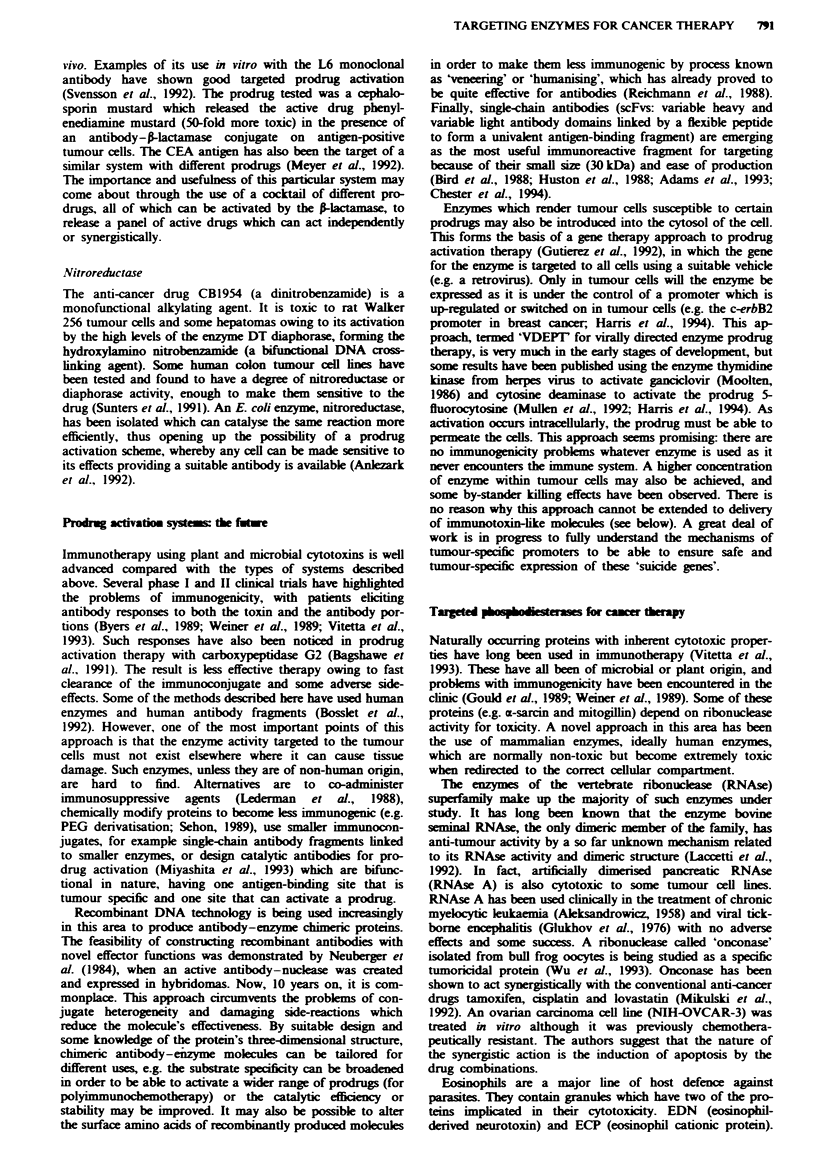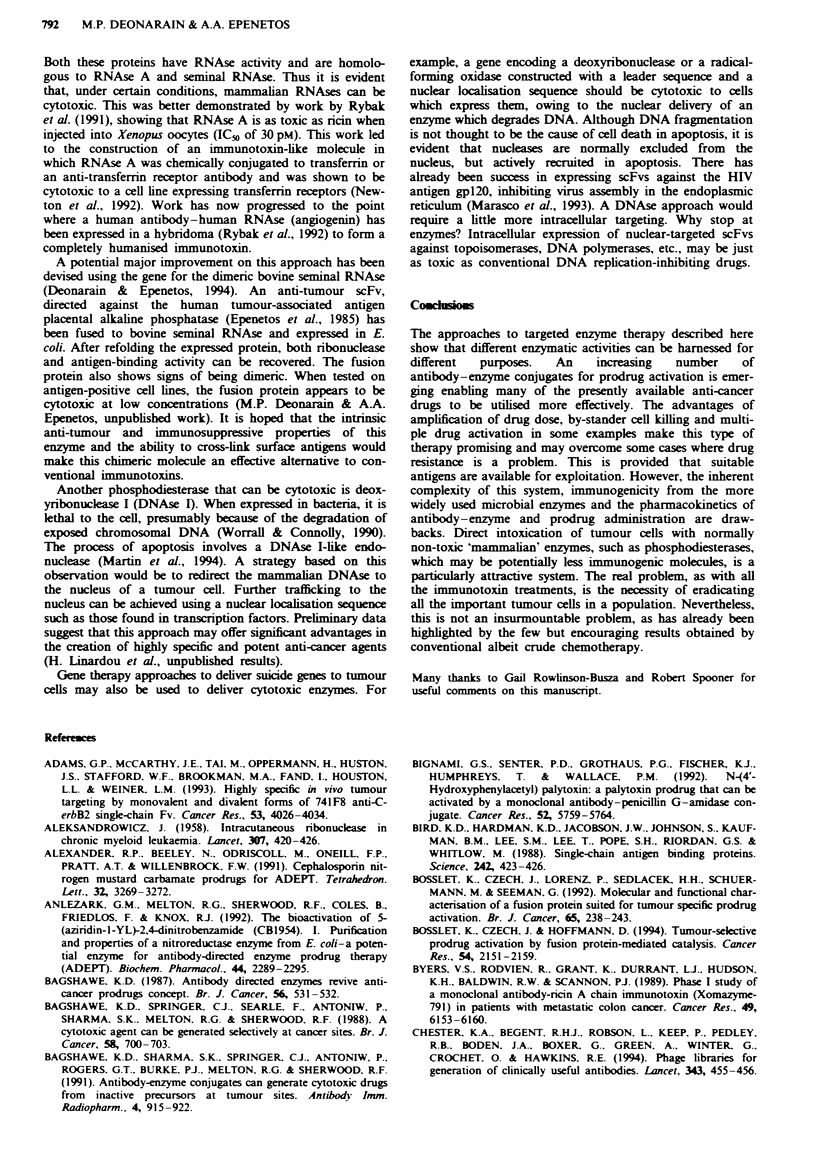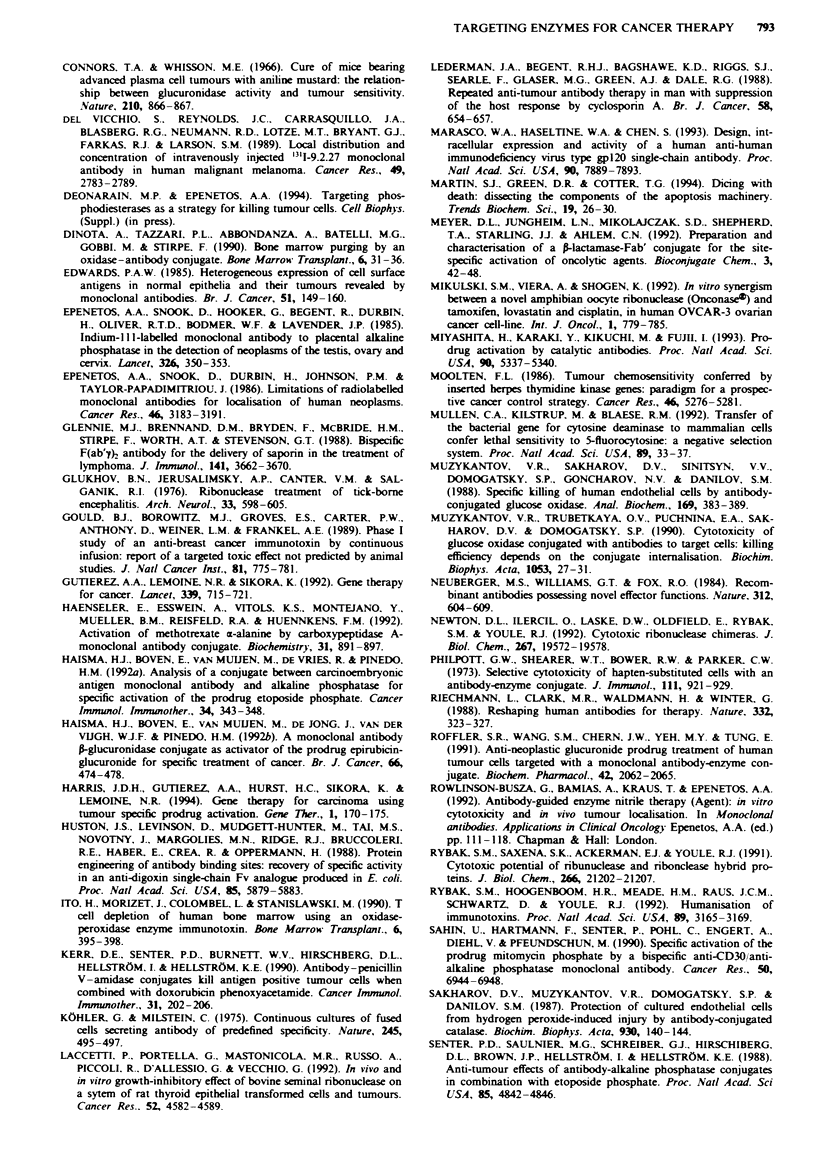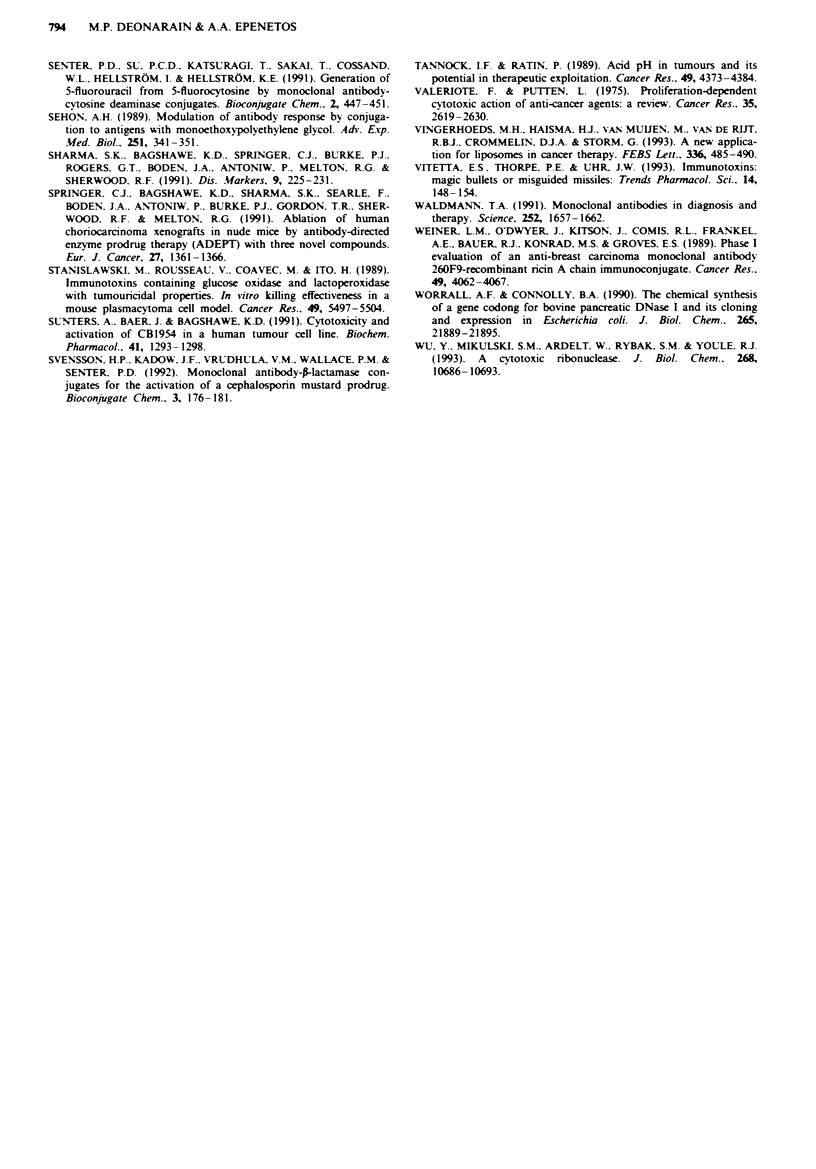# Targeting enzymes for cancer therapy: old enzymes in new roles.

**DOI:** 10.1038/bjc.1994.400

**Published:** 1994-11

**Authors:** M. P. Deonarain, A. A. Epenetos

**Affiliations:** Tumour Targeting Laboratory, Royal Postgraduate Medical School, Hammersmith Hospital, London, UK.

## Abstract

Enzymes which traditionally have played no role in cell-directed cytotoxicity are finding their way into schemes for prodrug activation and immunotoxins owing to such useful enzymatic activity. Alkaline phosphatase, carboxypeptidases, beta-glucosidases and beta-lactamases among many others are being utilised to regenerate potent anti-cancer drugs or toxic small molecules from precursors in a bid to enhance their activity in tumours. These prodrug activation systems require the pretargeting of the enzyme to the surface of a tumour cell, usually by an antibody or its immunoreactive fragment. A recent novel approach proposes the intracellular delivery of appropriate enzymes, such as phosphodiesterases, to particular cellular compartments. There, enzyme activity can cause substantive damage resulting in cell death. Cell targeting of mammalian phosphodiesterase promises to improve upon conventional immunotoxins because of their increased cytotoxicity when targeted to the appropriate compartment and their expected lack of, or lower, immunogenicity in clinical use.


Br. J. Cancer (1994), 70, 786-794 © Macmillan Press Ltd., 1994

REVIEW

### Targeting enzymes for cancer therapy: old enzymes in new roles

M.P. Deonarain & A.A. Epenetos

*Tumour Targeting Laboratory, ICRF Oncology Unit, Royal Postgraduate Medical School, Hammersmith Hospital, Du Cane Road, London W12 0HS, UK.*

**Summary** Enzymes which traditionally have played no role in cell-directed cytotoxicity are finding their way into schemes for prodrug activation and immunotoxins owing to such useful enzymatic activity. Alkaline phosphatase, carboxypeptidases, β-glucosidases and β-lactamases among many others are being utilised to regenerate potent anti-cancer drugs or toxic small molecules from precursors in a bid to enhance their activity in tumours. These prodrug activation systems require the pretargeting of the enzyme to the surface of a tumour cell, usually by an antibody or its immunoreactive fragment. A recent novel approach proposes the intracellular delivery of appropriate enzymes, such as phosphodiesterases, to particular cellular compartments. There, enzyme activity can cause substantive damage resulting in cell death. Cell targeting of mammalian phosphodiesterases promises to improve upon conventional immunotoxins because of their increased cytotoxicity when targeted to the appropriate compartment and their expected lack of, or lower, immunogenicity in clinical use,

The toxicities associated with conventional cancer chemotherapy arise primarily from the lack of specificity for tumour cells. Most of the presently available drugs are designed to be selectively toxic to rapidly dividing cells (Valeriote & Putten, 1975). This results in a low therapeutic index, which causes unacceptable damage to normal organs, limiting the drug dose that can be administered.

A variety of approaches are under development to improve the effectiveness and tumour cell specificity of cancer treatment. Many such methods involve monoclonal antibodies and offer attractive means of directing to a tumour toxic agents such as drugs, radioisotopes, protein cytotoxins, cytokines, effector cells of the immune system or, as in this review, enzymes.

The concept of targeting enzymes to tumour cells can be divided into two quite different approaches (Figure 1). Directing enzymes which catalyse precursor drug conversion into active drug at the site of a tumour [known as prodrug activation therapy, antibody-directed enzyme prodrug therapy (ADEPT) or antibody-directed catalysis (ADC)] entails using whole antibodies (or their fragments) raised against tumour-associated antigens to deliver enzymes specifically to tumour cells and catalyse the desired conversion reaction *in situ*. The prodrug is designed to be a substrate for the chosen enzyme. This work is now well advanced, with many examples of systems already tested in preclinical models and some entering clinical trials.

The second and possibly more novel approach is that of targeting mammalian enzymes, which are usually non-cytotoxic and non-immunogenic, internally to a particular cell compartment where they can catalyse a reaction resulting in cell death. This method is analogous to that of immunotoxins, naturally occurring cytotoxic proteins of bacterial, plant or fungal origin that are directed inside target cells and cause death as a result of a specific, potent chemical reaction (for example, the ribosylation of elongation factor 2 by *Pseudomonas* exotoxin).

### Prodrug activation systems

Tumour antigens which do not readily internalise or cycle to and from the cell surface lend themselves as targets for prodrug activation systems. Such antigens are human carcinoembryonic antigen (CEA) and human chorionic gonadotropin (hCG).

Monoclonal antibodies remain the choice vehicle for targeting, because of their high specificity as well as ease of isolation and manipulation (Köhler & Milstein, 1975; Waldmann, 1991). However, experiments using radiolabelled antibodies have shown that distribution of whole or even fragments of antibodies is not always optimal. Owing to their large size (150 kDa for IgG), antibodies penetrate slowly into solid tumour masses and are restricted to areas in the tumour which are near blood vessels (Del Vicchio *et al*., 1989). Antibodies are cleared slowly from the circulation, resulting in a high background level. A further limitation is that not all the cells in the tumour are detected. This is because of the heterogeneous expression of tumour-associated antigens, that is the existence of a population of antigen-negative tumour cells (Edwards, 1985). As monoclonal antibodies entered clinic use it became apparent that 100-1,000 times less antibody is taken up by tumours than in animal xenograft studies (Epenetos *et al*., 1986).

These observations gave rise to the concept of directed prodrug activation therapy (Bagshawe, 1987). By way of a monoclonal antibody (or any other cell-specific ligand), an enzyme can be accumulated within a tumour at a significantly higher concentration than in other tissues. The long half-life of this non-toxic molecule would allow maximal loading at the tumour site without significant background toxicity. The systemic administration of a suitably designed prodrug would generate a small, highly toxic drug within the tumour which is able to diffuse into the mass. Such drug molecules would kill tumour cells inaccessible to larger molecules, and neighbouring antigen-negative tumour cells would also be killed (by-stander effect), overcoming the problems of poor penetration and antigen heterogeneity. Since enzymes are catalytic proteins with high turnover, many active drug molecules would be generated by one enzyme molecule, thereby amplifying the number of toxic molecules at the site of the tumour, compensating for the low initial uptake. Some of the more extensively studied enzyme-mediated prodrug activation schemes (Table I) are described below. All the systems described except the β-glucuronidase fusion of Bosslet *et al*. (1992) involve chemical conjugation of the antibody and enzyme partners using stable non-reducible linkages. Each of these schemes has its strengths and limitations.

Correspondence: M.P. Deonarain.
Received 6 April 1994; and in revised form 28 June 1994.

**Figure 1** Alternative approaches to targeted enzyme therapy. **a**, Enzymes can be localised to the surface of the target cell and activate systemically administered prodrug. The toxic drug can then diffuse inside the tumour. **b**, Certain cell-bound molecules are taken into the cell by antigens and can be used to introduce enzymes, with toxic activities, into cellular compartments, e.g. ribonuclease activity in the cytosol.

### Generation of highly reactive toxic species *in situ*

Reduced oxygen species have been shown to participate in the anti-cancer action of chemotherapeutic agents, radiation therapy and host cell defence mechanisms. The molecules implicated are peroxide, superoxide and hydroxyl radicals. These and other small, toxic species such as cyanide have been incorporated into prodrug activation systems.

### *Oxidases*

The first example of targeted enzyme therapy was demonstrated by Philpott *et al.* (1973), who chemically conjugated glucose oxidase, which generates peroxide from glucose, to a polyclonal anti-hapten antibody. The conjugate was tested on hapten-substituted cells, but was not significantly cytotoxic on the addition of glucose. This may have been because of the low enzymatic activity of the conjugate or the lack of sensitivity of the cell line, since other workers have had more success with this particular system (Stanislawski *et al.*, 1989). However, when this sytem was coupled to lactoperoxidase and iodine, a marked increase in cellular toxicity was observed. Thus was demonstrated immunospecific cellular iodination, with cytotoxic effects.

The glucose oxidase system has also been investigated by Muzykantov *et al.* (1988) using polyclonal and monoclonal antibodies. Significant cytotoxicity was observed using a polyclonal antibody preparation, but studies using specific

**Table I** List of the prodrug activation systems currently under development, with the antigens and antibodies used. See text for references

| *Antibody/antigen* | *Enzyme* | *Prodrug* | *Active drug* |
|---|---|---|---|
| MAb 097/CDw52 pan-leucocyte) | Glucose oxidase and lactoperoxidase | Glucose (dextrose) sodium iodide | Hydrogen peroxide and toxic iodine species |
| MAb 8A/plasma cell-associated antigen | Xanthine oxidase | Xanthine, hypoxanthine | Hydrogen peroxide, hydroxyl and oxygen radicals |
| MAbs W14A and SB10/ chorionic gonadotropin | Carboxypeptidase G2 | Benzoic acid mustards-glutamic acid | Benzoic acid mustards |
| MAb L6 (against a carbohydrate antigen on human carcinomas) | Alkaline phosphatase | Etoposide phosphate, doxorubicin phosphate, mitomycin phosphate phenol mustard phosphate | Etoposide, doxorubicin, mitomycin Phenol mustard |
| MAb BW431/26/ carcinoembryonic antigen (CEA) | Alkaline phosphatase | Etoposide phosphate | Etoposide |
| MAb KS1/4/UCLA-P3 human lung adenocarcinoma | Carboxypeptidase A | Methotrexate-alanine | Methotrexate |
| MAb L6 (see above) | Cytosine deaminase | 5-Fluorocytosine (5-FC) | 5-Fluorouracil (5FU) |
| MAb L6 (see above) | Penicillin amidase | Doxorubicin-phenoxyacetamide Melphalan-phenoxyacetamide Palytoxin-4 hydroxyphenoxyacetamide | Doxorubicin Melphalan Palytoxin |
| MAb H17E2/ anti-placental alkaline phosphatase | β-Glucosidase | Amygdalin | Cyanide |
| MAb 323/A3 (against a pan-carcinoma membrane glycoprotein | β-Glucuronidase | Epirubicin-glucoronide | Epirubicin |
| MAb 12.8/ colon carcinoma | β-Glucuronidase | Phenol mustard glucoronide | Phenol mustard |
| MAb BW431/26, Fa anti-CEA | β-Glucuronidase | Daunomycin-glucoronide Adriamycin-glucoronide | Daunomycin Adriamycin |
| MAb L6 [see above, $F(ab')_2$] | β-Lactamase | Phenylenediamine mustard cephalosporin | Phenylenediamine mustard |
| No antibody in study | β-Lactamase | Vinca-cephalosporin | Vinca alkaloid |
| | β-Lactamase | Nitrogen mustard-cephalosporin | Nitrogen mustard |
| | Nitroreductase | CB1954 (5-aziridin 2,4-dinitrobenzamidine) | 5-aziridin 2,4-hydroxyamino 2-nitrobenzamidine |

monoclonal antibodies have suggested that the more potent mode of action could be due to its internalisation, the conjugate acting in an immunotoxin-like manner (see below) by generating intracellular peroxides, rather than activating prodrugs at the cell surface (Muzykantov *et al.*, 1990).

The enzyme xanthine oxidase generates reduced oxygen species from xanthine or hypoxanthine. The potency of the free radicals formed was enhanced by the presence of chelated iron. When conjugated to a monoclonal antibody against a human plasma cell-associated antigen it formed an effective prodrug activation system which was selectively cytotoxic to plasma cells but was non-toxic to myeloid precursors (Dinota *et al.*, 1990). Its usefulness was shown in the ability to purge neoplastic plasma cells from bone marrow suspensions *in vitro*, sparing the stem cells for autologous bone marrow transplantation. Similar systems to deplete T cells from bone marrow have been developed using the glucose oxidase/lactoperoxidase system (Ito *et al.*, 1990). Unfortunately, these types of systems would find little use *in vivo* owing to the presence of neutralising serum enzymes such as catalase and superoxide dismutase. Alternatively, such neutralising properties have been put to use by targeting antibody-catalase conjugates to cells in order to protect them by positive selection (Sakharov *et al.*, 1987).

Studies on oxidative enzyme conjugates have shown that very few neighbouring cells are killed by their action, probably explaining their success at purging specific populations of cells from a mixture. This may be because of extremely short half-life of the toxic molecules produced. In another system, a very small toxic species is generated by prodrug activation which is not affected by the neutralising action of serum enzymes and may have a half-life long enough to have a bystander killing effect. This is the cyanide generation system devised by Rowlinson-Busza *et al.* (1992) (antibody-guided enzyme nitrile therapy).

*Antibody-guided enzyme nitrile therapy*

This system, known as AGENT, is based upon the enzyme β-glucosidase conjugated to a tumour-specific monoclonal antibody. The β-glucosidase from sweet almonds has a broad substrate specificity enabling it to catalyse the conversion of a disaccharide, amygdalin to glucose, benzaldehyde and hydrogen cyanide (Figure 2). Amygdalin has been used, ineffectively, as an anti-cancer treatment under the name Laetrile. Its poor activity was due to the slow, untargeted release of the toxic moiety, cyanide. Human tumours do not express a β-glucosidase which is capable of activating amygdalin. With the targeted system, cyanide can diffuse into tumour and surrounding cells and kill them by inhibiting mitochondrial respiration. The work has already produced encouraging results: cell-bound antibody-β-glucosidase conjugate can enhance the cytotoxicity of amygdalin to that of the level of cyanide alone *in vitro*. This results in a 1,000-fold enhancement of toxicity (Rowlinson-Busza *et al.*, 1992). Cyanide is not as toxic as most conventional drugs ($IC_{50}$ 200μM), but generation of a high enough concentration within the tumour should have beneficial results. These high doses can be realised, as amygdalin itself is non-toxic.

A paradigm of the prodrug activation system that can be tested easily in the clinic is that of intravesicle tretment of bladder cancer: high concentrations of amygdalin can be administered and the antigen is easily accessible.

The genes for specific cyanogenic β-glucosidases have been cloned, making it feasible to construct recombinant antibody-β-glucosidase fusion proteins. Recombinant creation of molecules suitable for prodrug activation is a more exact method than chemical conjugation, because of the unwanted side-reactions which occur upon chemical cross-linking. Also, genetic manipulation can allow molecules to be altered and tailored to the required function, for example decreasing the size of the fusion protein by using single-chain antibodies or altering substrate specificity by active-site protein engineering. The recombinantly produced molecule should have a much higher activity, as was found for the β-glucuronidase fusion protein (Bosslet *et al.*, 1992).

**Regeneration of conventional anti-cancer drugs *in situ***

A great deal of effort has been placed into improving the action of conventional chemotherapy drugs such as

Figure 2 Examples of the structures of three of the prodrugs reviewed and their enzymic conversion to active drugs.

alkylating agents and DNA synthesis inhibitors, by aiming to generate them at high concentrations in tumour cells in preference to other organ sites. The similarities between normal and neoplastic cells have made it difficult to identify specific tumour-expressed enzymes which may be able to activate prodrugs. Subcutaneous placement of encapsulated enzymes into tumours, followed by the administration of the prodrug, has been one attempt (e.g. cytosine deaminase). This, of course, is ineffective against disseminated disease. Antibody delivery of a myriad of enzymes has made it possible to activate many different prodrugs, which are relatively non-toxic forms of pre-existing anti-cancer drugs. The derivatisation of an active drug can lower its toxicity in a number of ways. For example, the addition of a charged group can reduce its ability to permeate cells, the addition of a chemical group may reduce the reactivity of the drug or the alteration of the drug may reduce its affinity for the cellular target. With this approach, correct timing of antibody–enzyme and prodrug administration can lead to both a high level of cytotoxicity at all accessible tumour deposits systemically and low toxicity elsewhere.

*Alkaline phosphatase*

Many established chemotherapeutic drugs have been phosphorylated to form much less active prodrugs suitable for regeneration by the enzyme alkaline phosphatase, which hydrolytically removes phosphates (Figure 2). The phosphorylated version of the first drug to be used, etoposide, is more than 100-fold less toxic to a human carcinoma cell line (Senter *et al.*, 1988). When alkaline phosphatase was directed to tumour cells *in vitro* and *in vivo* by the tumour-specific monoclonal antibody L6, significant immunospecific anti-tumour activity was seen. The alkaline phosphatase used here, however, was from calf intestine. It is not ideal for therapy, as unacceptably high levels of alkaline phosphatase accumulate in many tissues, causing a great deal of prodrug to be prematurely activated. Similar work on this system using an anti-CEA antibody has shown that prolonged exposure to the etoposide–phosphate prodrug is almost as toxic as the activated drug (Haisma *et al.*, 1992*a*). Other systems have sought to overcome this major problem, and it is likely that the use of alkaline phosphatase, although successful in demonstrating the concept, will be superseded by the use of better suited enzymes.

Bispecific antibodies have been used successfully to deliver cytotoxins such as saporin to tumour cells (Glennie *et al.*, 1988). This approach has also been incorporated in a prodrug activation system to deliver alkaline phosphatase to a Hodgkin's disease-derived tumour cell line (Sahin *et al.*, 1990). An anti-alkaline phosphatase/anti CD30 bispecific antibody, expressed from a hybrid hybridoma, was preincubated with the enzyme before the whole assembly was tested on antigen-positive cells. The prodrug, mitomycin phosphate, was activated to levels similar to those seen with the chemical conjugate approach. This method, which can be applied to other enzymes, eliminates the need for chemical cross-linking, which can impair both the antigen binding and enzymatic activities.

*Carboxypeptidase G2*

One of the key features of the prodrug activation approach should be that the enzyme does not occur naturally in normal tissues. Prokaryotes express many such unique enzymes and were the source of the glutamic acid exopeptidase, carboxypeptidase G2 (from *Pseudomonas*), which forms the basis of the approach pioneered by Bagshawe *et al.* (1988). This enzyme has been chemically linked to the $F(ab')_2$ fragments of an anti-hCG or anti-CEA antibody and used to activate glutamic acid derivatives of various nitrogen mustards (Figure 2). The prodrugs were approximately 100-fold less toxic than the parent drugs. This work is now well advanced, and a substantial amount of *in vivo* preclinical work has been carried out. In a subcutaneous choriocarcinoma mouse xenograft model, potent anti-tumour activity was observed in mice which had received the conjugate followed by the prodrug under different treatment regimens. The best one, resulting in eradication of a tumour xenograft, was obtained when 3 × 10 mg per mouse doses of prodrug were administered 72 h after 50 enzyme units of antibody–enzyme conjugate was injected (Springer *et al.*, 1991). The tumor was previously shown to be resistant to conventional chemotherapy (e.g. methotrexate and actinomycin D). Measurement of the levels of enzyme, prodrug and drug in tumours and normal tissues has shown that the tumour had the highest ratio of active drug to prodrug, implying that site-specific activation is occurring. However, there was a significantly higher absolute amount of active drug in the liver, kidney and blood, suggesting that the prodrug was being activated elsewhere. A three-phased system has been developed to combat the high background levels, by injecting a clearing antibody (Sharma *et al.*, 1991). This takes the form of a galactosylated anti-enzyme antibody antibody (raised against the active site of the enzyme). This large antibody 'mops up' circulating conjugate and is not as accessible to the tumour as the initial enzyme conjugate. It is then cleared rapidly by the liver via galactose receptors and is not active in that organ. This allows the prodrug to be administered earlier (within 24 h) with similar results as before. This ADEPT approach is under further development with many prodrug/drug combinations being tested for efficacy.

*Carboxypeptidase A*

The anti-folate drug methotrexate can be derivatised by the addition of alanine to its carboxylic acid group, resulting in a lowering of its $ID_{50}$ by almost 200-fold (to $8.8 \times 10^{-5}$ M). Bovine pancreas carboxypeptidase A can remove alanine residues from these molecules, enabling the active drug to be internalised by folate transport systems. This enzyme was coupled to an antibody directed against a lung adenocarcinoma cell line (UCLA-P3) in a prodrug activation system (Haenseler *et al.*, 1992). However the antibody–enzyme conjugate could only reactivate the prodrug moderately (6-fold) and, as with many of these systems, high cytotoxicity was found with prolonged exposure to the prodrug. The authors suggest that cell-surface peptidases, a slow uptake or folate starvation caused by the competing prodrug may be the cause. Other derivitised folate antagonists may give better results in future.

*β-Glucuronidase*

Mice bearing well-established PC5 plasma tumours were found to express high levels of β-glucuronidase, which were able to activate aniline mustard prodrugs derivitised (*in vivo* by the liver) with β-glucopyranoside (Connors & Whisson, 1966). This strategy proved ineffective in humans because of the low levels of β-glucuronidase. This led the way to systems involving targeted β-glucuronidases (GUS). The *Escherichia coli* and human form of the enzyme have both been used. A chemical conjugate of the *E. coli* enzyme to a pan-carcinoma antibody is able to activate the glucuronide form of the drug epirubicin (Haisma *et al.*, 1992*b*). Epirubicin–glucuronide (epi–glu) is a metabolically inactivated form of epirubicin isolated from the urine of patients treated with the conventional anti-cancer drug (1 mg can be purified per patient). It is 100- to 1,000-fold less toxic than the metabolically active drug, a more impressive value than most of the prodrug examples described here ($IC_{50}$ of epi–glu is 20μM). The *E. coli* enzyme has also been used in conjunction with aniline mustard prodrugs, which are over 500-fold less toxic than the parent drug (Roffler *et al.*, 1991). It is also suggested that in this system the liver enzyme UDP gluconosyltransferase may convert circulating active drug back into prodrug. Overall, these systems, although in early developmental stages, seem to have many advantages over the other approaches: the endogenous enzyme is present intracellularly and not exposed to the prodrug and the prodrugs are 10–100 times more non-toxic than other prodrug/drug combinations. Also, the pH microenvironment around the tumour is acid to neutral (Tannock & Ratin, 1989) and not optimal for enzymes such as alkaline phosphatase, but suitable for GUS (*E. coli* GUS pH optimum is 6.8, human GUS pH optimum is 5.4). Lastly, the use of the human enzyme may reduce immunogenicity permitting repeated administration. A human anti-CEA Fab'–human GUS fusion protein has been constructed and expressed in baby hamster kidney transfectomas (Bosslet *et al.*, 1992). Some impressive results in tumour xenograft studies have been achieved using this fusion protein and doxorubicin–glucuronide as a prodrug (Bosslet *et al.*, 1994). This report showed that very high tumour–normal organ ratios of the antibody–enzyme fusion protein could be achieved by 7 days, allowing specific prodrug activation. Significant tumour growth delays of around 30 days were seen in this system.

It has been reported that more effective prodrug activation can be achieved *in vitro* using liposomes. In this case large aggregates consisting of a liposome with some 400 Fab' fragments and 20 β-glucoronidases has been made (Vingerhoeds *et al.*, 1993). Whether this assembly is effective *in vivo* despite its large size remains to be seen.

*Cytosine deaminase*

Fungi are intoxicated by the drug 5-fluorocytosine (5-FC), through its intracellular conversion to 5-fluorouracil (5-FU) by the fungus-specific enzyme cytosine deaminase (CDase). This forms the basis of an antifungal treatment and appears to be a suitable drug for a prodrug activation system. The 5-FC prodrug is non-toxic at up to 200 mM, whereas the 5-FU drug has an $IC_{50}$ of 20 μM *in vitro*. Although not as toxic as some anti-cancer drugs (e.g. methotrexate $IC_{50}$ is 0.05 μM) the low toxicity and established clinical use of 5-FC make it an attractive molecule which may be effective if generated at high enough concentrations. Unfortunately, many cancers develop drug resistance to 5-FU. Work using the L6 antibody and CDase purified from bakers' yeast has shown immunospecific cytotoxicity (Senter *et al.*, 1991). This enzyme has also been the subject of a gene therapy approach to targeting enzymes (see below).

*Penicillin amidase*

This enzyme is used industrially to hydrolyse the phenoxyacetamide group from penicillin V to yield 6-aminopenicillanic acid. The anti-cancer drugs doxorubicin and melphalan have been derivatised with *p*-hydroxyphenoxyacetamide to form substrates (DPO and MPO respectively) which are readily converted to the active drug by the enzyme (Kerr *et al.*, 1990). The DPO prodrug is 80-fold less toxic than doxorubicin ($IC_{50}$ 30 nM) whereas the MPO prodrug is more than 1,000-fold less toxic than melphalan against tumour cell-lines. However, especially in the case of the MPO prodrug, the antibody–enzyme conjugate was not able to liberate the active drug from the prodrug effectively in order to produce a cytotoxic effect, suggesting that the enzyme catalysed the reaction too slowly to be useful. This highlights the importance of having an enzyme with a high turnover in order to generate high concentrations of active drug. Penicillin-G-amidase has also been used in this context to activate the prodrug palytoxin (Bignami *et al.*, 1992).

*β-Lactamase*

The β-lactamases from *E. coli*, *Enterobacter cloacae* and *Bacillus* have good cephalosporinase activity and are able to accommodate a wide range of 3'substituents in the active site yet retain the ability to cleave the β-lactam ring. This activity enables the enzyme to release the cephalosporin group from a wide range of prodrugs, including nitrogen mustard and vinca alkaloid derivatives (Alexander *et al.*, 1991). In addition, eukaryotic cells have no such enzyme or related enzyme which could cause unfavourable activation of prodrugs *in*

*vivo*. Examples of its use *in vitro* with the L6 monoclonal antibody have shown good targeted prodrug activation (Svensson *et al*., 1992). The prodrug tested was a cephalosporin mustard which released the active drug phenylenediamine mustard (50-fold more toxic) in the presence of an antibody–β-lactamase conjugate on antigen-positive tumour cells. The CEA antigen has also been the target of a similar system with different prodrugs (Meyer *et al*., 1992). The importance and usefulness of this particular system may come about through the use of a cocktail of different prodrugs, all of which can be activated by the β-lactamase, to release a panel of active drugs which can act independently or synergistically.

### *Nitroreductase*

The anti-cancer drug CB1954 (a dinitrobenzamide) is a monofunctional alkylating agent. It is toxic to rat Walker 256 tumour cells and some hepatomas owing to its activation by the high levels of the enzyme DT diaphorase, forming the hydroxylamino nitrobenzamide (a bifunctional DNA cross-linking agent). Some human colon tumour cell lines have been tested and found to have a degree of nitroreductase or diaphorase activity, enough to make them sensitive to the drug (Sunters *et al*., 1991). An *E. coli* enzyme, nitroreductase, has been isolated which can catalyse the same reaction more efficiently, thus opening up the possibility of a prodrug activation scheme, whereby any cell can be made sensitive to its effects providing a suitable antibody is available (Anlezark *et al*., 1992).

### Prodrug activation systems: the future

Immunotherapy using plant and microbial cytotoxins is well advanced compared with the types of systems described above. Several phase I and II clinical trials have highlighted the problems of immunogenicity, with patients eliciting antibody responses to both the toxin and the antibody portions (Byers *et al*., 1989; Weiner *et al*., 1989; Vitetta *et al*., 1993). Such responses have also been noticed in prodrug activation therapy with carboxypeptidase G2 (Bagshawe *et al*., 1991). The result is less effective therapy owing to fast clearance of the immunoconjugate and some adverse side-effects. Some of the methods described here have used human enzymes and human antibody fragments (Bosslet *et al*., 1992). However, one of the most important points of this approach is that the enzyme activity targeted to the tumour cells must not exist elsewhere where it can cause tissue damage. Such enzymes, unless they are of non-human origin, are hard to find. Alternatives are to co-administer immunosuppressive agents (Lederman *et al*., 1988), chemically modify proteins to become less immunogenic (e.g. PEG derivatisation; Sehon, 1989), use smaller immunoconjugates, for example single-chain antibody fragments linked to smaller enzymes, or design catalytic antibodies for prodrug activation (Miyashita *et al*., 1993) which are bifunctional in nature, having one antigen-binding site that is tumour specific and one site that can activate a prodrug.

Recombinant DNA technology is being used increasingly in this area to produce antibody–enzyme chimeric proteins. The feasibility of constructing recombinant antibodies with novel effector functions was demonstrated by Neuberger *et al*. (1984), when an active antibody–nuclease was created and expressed in hybridomas. Now, 10 years on, it is commonplace. This approach circumvents the problems of conjugate heterogeneity and damaging side-reactions which reduce the molecule's effectiveness. By suitable design and some knowledge of the protein's three-dimensional structure, chimeric antibody–enzyme molecules can be tailored for different uses, e.g. the substrate specificity can be broadened in order to be able to activate a wider range of prodrugs (for polyimmunochemotherapy) or the catalytic efficiency or stability may be improved. It may also be possible to alter the surface amino acids of recombinantly produced molecules in order to make them less immunogenic by process known as 'veneering' or 'humanising', which has already proved to be quite effective for antibodies (Reichmann *et al*., 1988). Finally, single-chain antibodies (scFvs: variable heavy and variable light antibody domains linked by a flexible peptide to form a univalent antigen-binding fragment) are emerging as the most useful immunoreactive fragment for targeting because of their small size (30 kDa) and ease of production (Bird *et al*., 1988; Huston *et al*., 1988; Adams *et al*., 1993; Chester *et al*., 1994).

Enzymes which render tumour cells susceptible to certain prodrugs may also be introduced into the cytosol of the cell. This forms the basis of a gene therapy approach to prodrug activation therapy (Gutierez *et al*., 1992), in which the gene for the enzyme is targeted to all cells using a suitable vehicle (e.g. a retrovirus). Only in tumour cells will the enzyme be expressed as it is under the control of a promoter which is up-regulated or switched on in tumour cells (e.g. the c-*erb*B2 promoter in breast cancer; Harris *et al*., 1994). This approach, termed 'VDEPT' for virally directed enzyme prodrug therapy, is very much in the early stages of development, but some results have been published using the enzyme thymidine kinase from herpes virus to activate ganciclovir (Moolten, 1986) and cytosine deaminase to activate the prodrug 5-fluorocytosine (Mullen *et al*., 1992; Harris *et al*., 1994). As activation occurs intracellularly, the prodrug must be able to permeate the cells. This approach seems promising: there are no immunogenicity problems whatever enzyme is used as it never encounters the immune system. A higher concentration of enzyme within tumour cells may also be achieved, and some by-stander killing effects have been observed. There is no reason why this approach cannot be extended to delivery of immunotoxin-like molecules (see below). A great deal of work is in progress to fully understand the mechanisms of tumour-specific promoters to be able to ensure safe and tumour-specific expression of these 'suicide genes'.

### Targeted phosphodiesterases for cancer therapy

Naturally occurring proteins with inherent cytotoxic properties have long been used in immunotherapy (Vitetta *et al*., 1993). These have all been of microbial or plant origin, and problems with immunogenicity have been encountered in the clinic (Gould *et al*., 1989; Weiner *et al*., 1989). Some of these proteins (e.g. α-sarcin and mitogillin) depend on ribonuclease activity for toxicity. A novel approach in this area has been the use of mammalian enzymes, ideally human enzymes, which are normally non-toxic but become extremely toxic when redirected to the correct cellular compartment.

The enzymes of the vertebrate ribonuclease (RNAse) superfamily make up the majority of such enzymes under study. It has long been known that the enzyme bovine seminal RNAse, the only dimeric member of the family, has anti-tumour activity by a so far unknown mechanism related to its RNAse activity and dimeric structure (Laccetti *et al*., 1992). In fact, artificially dimerised pancreatic RNAse (RNAse A) is also cytotoxic to some tumour cell lines. RNAse A has been used clinically in the treatment of chronic myelocytic leukaemia (Aleksandrowicz, 1958) and viral tick-borne encephalitis (Glukhov *et al*., 1976) with no adverse effects and some success. A ribonuclease called 'onconase' isolated from bull frog oocytes is being studied as a specific tumoricidal protein (Wu *et al*., 1993). Onconase has been shown to act synergistically with the conventional anti-cancer drugs tamoxifen, cisplatin and lovastatin (Mikulski *et al*., 1992). An ovarian carcinoma cell line (NIH-OVCAR-3) was treated *in vitro* although it was previously chemotherapeutically resistant. The authors suggest that the nature of the synergistic action is the induction of apoptosis by the drug combinations.

Eosinophils are a major line of host defence against parasites. They contain granules which have two of the proteins implicated in their cytotoxicity. EDN (eosinophil-derived neurotoxin) and ECP (eosinophil cationic protein).

Both these proteins have RNAse activity and are homologous to RNAse A and seminal RNAse. Thus it is evident that, under certain conditions, mammalian RNAses can be cytotoxic. This was better demonstrated by work by Rybak *et al.* (1991), showing that RNAse A is as toxic as ricin when injected into *Xenopus* oocytes ($IC_{50}$ of 30 pM). This work led to the construction of an immunotoxin-like molecule in which RNAse A was chemically conjugated to transferrin or an anti-transferrin receptor antibody and was shown to be cytotoxic to a cell line expressing transferrin receptors (Newton *et al.*, 1992). Work has now progressed to the point where a human antibody–human RNAse (angiogenin) has been expressed in a hybridoma (Rybak *et al.*, 1992) to form a completely humanised immunotoxin.

A potential major improvement on this approach has been devised using the gene for the dimeric bovine seminal RNAse (Deonarain & Epenetos, 1994). An anti-tumour scFv, directed against the human tumour-associated antigen placental alkaline phosphatase (Epenetos *et al.*, 1985) has been fused to bovine seminal RNAse and expressed in *E. coli*. After refolding the expressed protein, both ribonuclease and antigen-binding activity can be recovered. The fusion protein also shows signs of being dimeric. When tested on antigen-positive cell lines, the fusion protein appears to be cytotoxic at low concentrations (M.P. Deonarain & A.A. Epenetos, unpublished work). It is hoped that the intrinsic anti-tumour and immunosuppressive properties of this enzyme and the ability to cross-link surface antigens would make this chimeric molecule an effective alternative to conventional immunotoxins.

Another phosphodiesterase that can be cytotoxic is deoxyribonuclease I (DNAse I). When expressed in bacteria, it is lethal to the cell, presumably because of the degradation of exposed chromosomal DNA (Worrall & Connolly, 1990). The process of apoptosis involves a DNAse I-like endonuclease (Martin *et al.*, 1994). A strategy based on this observation would be to redirect the mammalian DNAse to the nucleus of a tumour cell. Further trafficking to the nucleus can be achieved using a nuclear localisation sequence such as those found in transcription factors. Preliminary data suggest that this approach may offer significant advantages in the creation of highly specific and potent anti-cancer agents (H. Linardou *et al.*, unpublished results).

Gene therapy approaches to deliver suicide genes to tumour cells may also be used to deliver cytotoxic enzymes. For example, a gene encoding a deoxyribonuclease or a radical-forming oxidase constructed with a leader sequence and a nuclear localisation sequence should be cytotoxic to cells which express them, owing to the nuclear delivery of an enzyme which degrades DNA. Although DNA fragmentation is not thought to be the cause of cell death in apoptosis, it is evident that nucleases are normally excluded from the nucleus, but actively recruited in apoptosis. There has already been success in expressing scFvs against the HIV antigen gp120, inhibiting virus assembly in the endoplasmic reticulum (Marasco *et al.*, 1993). A DNAse approach would require a little more intracellular targeting. Why stop at enzymes? Intracellular expression of nuclear-targeted scFvs against topoisomerases, DNA polymerases, etc., may be just as toxic as conventional DNA replication-inhibiting drugs.

### Conclusions

The approaches to targeted enzyme therapy described here show that different enzymatic activities can be harnessed for different purposes. An increasing number of antibody–enzyme conjugates for prodrug activation is emerging enabling many of the presently available anti-cancer drugs to be utilised more effectively. The advantages of amplification of drug dose, by-stander cell killing and multiple drug activation in some examples make this type of therapy promising and may overcome some cases where drug resistance is a problem. This is provided that suitable antigens are available for exploitation. However, the inherent complexity of this system, immunogenicity from the more widely used microbial enzymes and the pharmacokinetics of antibody–enzyme and prodrug administration are drawbacks. Direct intoxication of tumour cells with normally non-toxic 'mammalian' enzymes, such as phosphodiesterases, which may be potentially less immunogenic molecules, is a particularly attractive system. The real problem, as with all the immunotoxin treatments, is the necessity of eradicating all the important tumour cells in a population. Nevertheless, this is not an insurmountable problem, as has already been highlighted by the few but encouraging results obtained by conventional albeit crude chemotherapy.

Many thanks to Gail Rowlinson-Busza and Robert Spooner for useful comments on this manuscript.

### References

ADAMS, G.P., McCARTHY, J.E., TAI, M., OPPERMANN, H., HUSTON, J.S., STAFFORD, W.F., BROOKMAN, M.A., FAND, I., HOUSTON, L.L. & WEINER, L.M. (1993). Highly specific *in vivo* tumour targeting by monovalent and divalent forms of 741F8 anti-C-*erb*B2 single-chain Fv. *Cancer Res.*, **53**, 4026–4034.

ALEKSANDROWICZ, J. (1958). Intracutaneous ribonuclease in chronic myeloid leukaemia. *Lancet*, **307**, 420–426.

ALEXANDER, R.P., BEELEY, N., ODRISCOLL, M., ONEILL, F.P., PRATT, A.T. & WILLENBROCK, F.W. (1991). Cephalosporin nitrogen mustard carbamate prodrugs for ADEPT. *Tetrahedron. Lett.*, **32**, 3269–3272.

ANLEZARK, G.M., MELTON, R.G., SHERWOOD, R.F., COLES, B., FRIEDLOS, F. & KNOX, R.J. (1992). The bioactivation of 5-(aziridin-1-YL)-2,4-dinitrobenzamide (CB1954). I. Purification and properties of a nitroreductase enzyme from *E. coli*–a potential enzyme for antibody-directed enzyme prodrug therapy (ADEPT). *Biochem. Pharmacol.*, **44**, 2289–2295.

BAGSHAWE, K.D. (1987). Antibody directed enzymes revive anticancer prodrugs concept. *Br. J. Cancer*, **56**, 531–532.

BAGSHAWE, K.D., SPRINGER, C.J., SEARLE, F., ANTONIW, P., SHARMA, S.K., MELTON, R.G. & SHERWOOD, R.F. (1988). A cytotoxic agent can be generated selectively at cancer sites. *Br. J. Cancer*, **58**, 700–703.

BAGSHAWE, K.D., SHARMA, S.K., SPRINGER, C.J., ANTONIW, P., ROGERS, G.T., BURKE, P.J., MELTON, R.G. & SHERWOOD, R.F. (1991). Antibody-enzyme conjugates can generate cytotoxic drugs from inactive precursors at tumour sites. *Antibody Imm. Radiopharm.*, **4**, 915–922.

BIGNAMI, G.S., SENTER, P.D., GROTHAUS, P.G., FISCHER, K.J., HUMPHREYS, T. & WALLACE, P.M. (1992). N-(4'-Hydroxyphenylacetyl) palytoxin: a palytoxin prodrug that can be activated by a monoclonal antibody–penicillin G–amidase conjugate. *Cancer Res.*, **52**, 5759–5764.

BIRD, K.D., HARDMAN, K.D., JACOBSON, J.W., JOHNSON, S., KAUFMAN, B.M., LEE, S.M., LEE, T., POPE, S.H., RIORDAN, G.S. & WHITLOW, M. (1988). Single-chain antigen binding proteins. *Science*, **242**, 423–426.

BOSSLET, K., CZECH, J., LORENZ, P., SEDLACEK, H.H., SCHUERMANN, M. & SEEMAN, G. (1992). Molecular and functional characterisation of a fusion protein suited for tumour specific prodrug activation. *Br. J. Cancer*, **65**, 238–243.

BOSSLET, K., CZECH, J. & HOFFMANN, D. (1994). Tumour-selective prodrug activation by fusion protein-mediated catalysis. *Cancer Res.*, **54**, 2151–2159.

BYERS, V.S., RODVIEN, R., GRANT, K., DURRANT, L.J., HUDSON, K.H., BALDWIN, R.W. & SCANNON, P.J. (1989). Phase I study of a monoclonal antibody-ricin A chain immunotoxin (Xomazyme-791) in patients with metastatic colon cancer. *Cancer Res.*, **49**, 6153–6160.

CHESTER, K.A., BEGENT, R.H.J., ROBSON, L., KEEP, P., PEDLEY, R.B., BODEN, J.A., BOXER, G., GREEN, A., WINTER, G., CROCHET, O. & HAWKINS, R.E. (1994). Phage libraries for generation of clinically useful antibodies. *Lancet*, **343**, 455–456.

CONNORS, T.A. & WHISSON, M.E. (1966). Cure of mice bearing advanced plasma cell tumours with aniline mustard: the relationship between glucuronidase activity and tumour sensitivity. *Nature*, **210**, 866–867.

DEL VICCHIO, S., REYNOLDS, J.C., CARRASQUILLO, J.A., BLASBERG, R.G., NEUMANN, R.D., LOTZE, M.T., BRYANT, G.J., FARKAS, R.J. & LARSON, S.M. (1989). Local distribution and concentration of intravenously injected $^{131}$I-9.2.27 monoclonal antibody in human malignant melanoma. *Cancer Res.*, **49**, 2783–2789.

DEONARAIN, M.P. & EPENETOS, A.A. (1994). Targeting phosphodiesterases as a strategy for killing tumour cells. *Cell Biophys.* (Suppl.) (in press).

DINOTA, A., TAZZARI, P.L., ABBONDANZA, A., BATELLI, M.G., GOBBI, M. & STIRPE, F. (1990). Bone marrow purging by an oxidase–antibody conjugate. *Bone Marrow Transplant.*, **6**, 31–36.

EDWARDS, P.A.W. (1985). Heterogeneous expression of cell surface antigens in normal epithelia and their tumours revealed by monoclonal antibodies. *Br. J. Cancer*, **51**, 149–160.

EPENETOS, A.A., SNOOK, D., HOOKER, G., BEGENT, R., DURBIN, H., OLIVER, R.T.D., BODMER, W.F. & LAVENDER, J.P. (1985). Indium-111-labelled monoclonal antibody to placental alkaline phosphatase in the detection of neoplasms of the testis, ovary and cervix. *Lancet*, **326**, 350–353.

EPENETOS, A.A., SNOOK, D., DURBIN, H., JOHNSON, P.M. & TAYLOR-PAPADIMITRIOU, J. (1986). Limitations of radiolabelled monoclonal antibodies for localisation of human neoplasms. *Cancer Res.*, **46**, 3183–3191.

GLENNIE, M.J., BRENNAND, D.M., BRYDEN, F., MCBRIDE, H.M., STIRPE, F., WORTH, A.T. & STEVENSON, G.T. (1988). Bispecific F(ab'γ)$_2$ antibody for the delivery of saporin in the treatment of lymphoma. *J. Immunol.*, **141**, 3662–3670.

GLUKHOV, B.N., JERUSALIMSKY, A.P., CANTER, V.M. & SALGANIK, R.I. (1976). Ribonuclease treatment of tick-borne encephalitis. *Arch. Neurol.*, **33**, 598–605.

GOULD, B.J., BOROWITZ, M.J., GROVES, E.S., CARTER, P.W., ANTHONY, D., WEINER, L.M. & FRANKEL, A.E. (1989). Phase I study of an anti-breast cancer immunotoxin by continuous infusion: report of a targeted toxic effect not predicted by animal studies. *J. Natl Cancer Inst.*, **81**, 775–781.

GUTIEREZ, A.A., LEMOINE, N.R. & SIKORA, K. (1992). Gene therapy for cancer. *Lancet*, **339**, 715–721.

HAENSELER, E., ESSWEIN, A., VITOLS, K.S., MONTEJANO, Y., MUELLER, B.M., REISFELD, R.A. & HUENNKENS, F.M. (1992). Activation of methotrexate α-alanine by carboxypeptidase A-monoclonal antibody conjugate. *Biochemistry*, **31**, 891–897.

HAISMA, H.J., BOVEN, E., VAN MUIJEN, M., DE VRIES, R. & PINEDO, H.M. (1992*a*). Analysis of a conjugate between carcinoembryonic antigen monoclonal antibody and alkaline phosphatase for specific activation of the prodrug etoposide phosphate. *Cancer Immunol. Immunother.*, **34**, 343–348.

HAISMA, H.J., BOVEN, E., VAN MUIJEN, M., DE JONG, J., VAN DER VIJGH, W.J.F. & PINEDO, H.M. (1992*b*). A monoclonal antibody β-glucuronidase conjugate as activator of the prodrug epirubicin-glucuronide for specific treatment of cancer. *Br. J. Cancer*, **66**, 474–478.

HARRIS, J.D.H., GUTIEREZ, A.A., HURST, H.C., SIKORA, K. & LEMOINE, N.R. (1994). Gene therapy for carcinoma using tumour specific prodrug activation. *Gene Ther.*, **1**, 170–175.

HUSTON, J.S., LEVINSON, D., MUDGETT-HUNTER, M., TAI, M.S., NOVOTNY, J., MARGOLIES, M.N., RIDGE, R.J., BRUCCOLERI, R.E., HABER, E., CREA, R. & OPPERMANN, H. (1988). Protein engineering of antibody binding sites: recovery of specific activity in an anti-digoxin single-chain Fv analogue produced in *E. coli*. *Proc. Natl Acad. Sci. USA*, **85**, 5879–5883.

ITO, H., MORIZET, J., COLOMBEL, L. & STANISLAWSKI, M. (1990). T cell depletion of human bone marrow using an oxidase-peroxidase enzyme immunotoxin. *Bone Marrow Transplant.*, **6**, 395–398.

KERR, D.E., SENTER, P.D., BURNETT, W.V., HIRSCHBERG, D.L., HELLSTRÖM, I. & HELLSTRÖM, K.E. (1990). Antibody–penicillin V–amidase conjugates kill antigen positive tumour cells when combined with doxorubicin phenoxyacetamide. *Cancer Immunol. Immunother.*, **31**, 202–206.

KÖHLER, G. & MILSTEIN, C. (1975). Continuous cultures of fused cells secreting antibody of predefined specificity. *Nature*, **245**, 495–497.

LACCETTI, P., PORTELLA, G., MASTONICOLA, M.R., RUSSO, A., PICCOLI, R., D'ALLESSIO, G. & VECCHIO, G. (1992). *In vivo* and *in vitro* growth-inhibitory effect of bovine seminal ribonuclease on a sytem of rat thyroid epithelial transformed cells and tumours. *Cancer Res.*, **52**, 4582–4589.

LEDERMAN, J.A., BEGENT, R.H.J., BAGSHAWE, K.D., RIGGS, S.J., SEARLE, F., GLASER, M.G., GREEN, A.J. & DALE, R.G. (1988). Repeated anti-tumour antibody therapy in man with suppression of the host response by cyclosporin A. *Br. J. Cancer*, **58**, 654–657.

MARASCO, W.A., HASELTINE, W.A. & CHEN, S. (1993). Design, intracellular expression and activity of a human anti-human immunodeficiency virus type gp120 single-chain antibody. *Proc. Natl Acad. Sci. USA*, **90**, 7889–7893.

MARTIN, S.J., GREEN, D.R. & COTTER, T.G. (1994). Dicing with death: dissecting the components of the apoptosis machinery. *Trends Biochem. Sci.*, **19**, 26–30.

MEYER, D.L., JUNGHEIM, L.N., MIKOLAJCZAK, S.D., SHEPHERD, T.A., STARLING, J.J. & AHLEM, C.N. (1992). Preparation and characterisation of a β-lactamase-Fab' conjugate for the site-specific activation of oncolytic agents. *Bioconjugate Chem.*, **3**, 42–48.

MIKULSKI, S.M., VIERA, A. & SHOGEN, K. (1992). *In vitro* synergism between a novel amphibian oocyte ribonuclease (Onconase®) and tamoxifen, lovastatin and cisplatin, in human OVCAR-3 ovarian cancer cell-line. *Int. J. Oncol.*, **1**, 779–785.

MIYASHITA, H., KARAKI, Y., KIKUCHI, M. & FUJII, I. (1993). Prodrug activation by catalytic antibodies. *Proc. Natl Acad. Sci. USA*, **90**, 5337–5340.

MOOLTEN, F.L. (1986). Tumour chemosensitivity conferred by inserted herpes thymidine kinase genes: paradigm for a prospective cancer control strategy. *Cancer Res.*, **46**, 5276–5281.

MULLEN, C.A., KILSTRUP, M. & BLAESE, R.M. (1992). Transfer of the bacterial gene for cytosine deaminase to mammalian cells confer lethal sensitivity to 5-fluorocytosine: a negative selection system. *Proc. Natl Acad. Sci. USA*, **89**, 33–37.

MUZYKANTOV, V.R., SAKHAROV, D.V., SINITSYN, V.V., DOMOGATSKY, S.P., GONCHAROV, N.V. & DANILOV, S.M. (1988). Specific killing of human endothelial cells by antibody-conjugated glucose oxidase. *Anal. Biochem.*, **169**, 383–389.

MUZYKANTOV, V.R., TRUBETKAYA, O.V., PUCHNINA, E.A., SAKHAROV, D.V. & DOMOGATSKY, S.P. (1990). Cytotoxicity of glucose oxidase conjugated with antibodies to target cells: killing efficiency depends on the conjugate internalisation. *Biochim. Biophys. Acta*, **1053**, 27–31.

NEUBERGER, M.S., WILLIAMS, G.T. & FOX, R.O. (1984). Recombinant antibodies possessing novel effector functions. *Nature*, **312**, 604–609.

NEWTON, D.L., ILERCIL, O., LASKE, D.W., OLDFIELD, E., RYBAK, S.M. & YOULE, R.J. (1992). Cytotoxic ribonuclease chimeras. *J. Biol. Chem.*, **267**, 19572–19578.

PHILPOTT, G.W., SHEARER, W.T., BOWER, R.W. & PARKER, C.W. (1973). Selective cytotoxicity of hapten-substituted cells with an antibody-enzyme conjugate. *J. Immunol.*, **111**, 921–929.

RIECHMANN, L., CLARK, M.R., WALDMANN, H. & WINTER, G. (1988). Reshaping human antibodies for therapy. *Nature*, **332**, 323–327.

ROFFLER, S.R., WANG, S.M., CHERN, J.W., YEH, M.Y. & TUNG, E. (1991). Anti-neoplastic glucuronide prodrug treatment of human tumour cells targeted with a monoclonal antibody-enzyme conjugate. *Biochem. Pharmacol.*, **42**, 2062–2065.

ROWLINSON-BUSZA, G., BAMIAS, A., KRAUS, T. & EPENETOS, A.A. (1992). Antibody-guided enzyme nitrile therapy (Agent): *in vitro* cytotoxicity and *in vivo* tumour localisation. In *Monoclonal antibodies. Applications in Clinical Oncology* Epenetos, A.A. (ed.) pp. 111–118. Chapman & Hall: London.

RYBAK, S.M., SAXENA, S.K., ACKERMAN, E.J. & YOULE, R.J. (1991). Cytotoxic potential of ribunuclease and ribonclease hybrid proteins. *J. Biol. Chem.*, **266**, 21202–21207.

RYBAK, S.M., HOOGENBOOM, H.R., MEADE, H.M., RAUS, J.C.M., SCHWARTZ, D. & YOULE, R.J. (1992). Humanisation of immunotoxins. *Proc. Natl Acad. Sci. USA*, **89**, 3165–3169.

SAHIN, U., HARTMANN, F., SENTER, P., POHL, C., ENGERT, A., DIEHL, V. & PFEUNDSCHUN, M. (1990). Specific activation of the prodrug mitomycin phosphate by a bispecific anti-CD30/anti-alkaline phosphatase monoclonal antibody. *Cancer Res.*, **50**, 6944–6948.

SAKHAROV, D.V., MUZYKANTOV, V.R., DOMOGATSKY, S.P. & DANILOV, S.M. (1987). Protection of cultured endothelial cells from hydrogen peroxide-induced injury by antibody-conjugated catalase. *Biochim. Biophys. Acta*, **930**, 140–144.

SENTER, P.D., SAULNIER, M.G., SCHREIBER, G.J., HIRSCHIBERG, D.L., BROWN, J.P., HELLSTRÖM, I. & HELLSTRÖM, K.E. (1988). Anti-tumour effects of antibody-alkaline phosphatase conjugates in combination with etoposide phosphate. *Proc. Natl Acad. Sci USA*, **85**, 4842–4846.

SENTER, P.D., SU, P.C.D., KATSURAGI, T., SAKAI, T., COSSAND, W.L., HELLSTRÖM, I. & HELLSTRÖM, K.E. (1991). Generation of 5-fluorouracil from 5-fluorocytosine by monoclonal antibody-cytosine deaminase conjugates. *Bioconjugate Chem.*, **2**, 447–451.

SEHON, A.H. (1989). Modulation of antibody response by conjugation to antigens with monoethoxypolyethylene glycol. *Adv. Exp. Med. Biol.*, **251**, 341–351.

SHARMA, S.K., BAGSHAWE, K.D., SPRINGER, C.J., BURKE, P.J., ROGERS, G.T., BODEN, J.A., ANTONIW, P., MELTON, R.G. & SHERWOOD, R.F. (1991). *Dis. Markers*, **9**, 225–231.

SPRINGER, C.J., BAGSHAWE, K.D., SHARMA, S.K., SEARLE, F., BODEN, J.A., ANTONIW, P., BURKE, P.J., GORDON, T.R., SHERWOOD, R.F. & MELTON, R.G. (1991). Ablation of human choriocarcinoma xenografts in nude mice by antibody-directed enzyme prodrug therapy (ADEPT) with three novel compounds. *Eur. J. Cancer*, **27**, 1361–1366.

STANISLAWSKI, M., ROUSSEAU, V., COAVEC, M. & ITO, H. (1989). Immunotoxins containing glucose oxidase and lactoperoxidase with tumouricidal properties. *In vitro* killing effectiveness in a mouse plasmacytoma cell model. *Cancer Res.*, **49**, 5497–5504.

SUNTERS, A., BAER, J. & BAGSHAWE, K.D. (1991). Cytotoxicity and activation of CB1954 in a human tumour cell line. *Biochem. Pharmacol.*, **41**, 1293–1298.

SVENSSON, H.P., KADOW, J.F., VRUDHULA, V.M., WALLACE, P.M. & SENTER, P.D. (1992). Monoclonal antibody-β-lactamase conjugates for the activation of a cephalosporin mustard prodrug. *Bioconjugate Chem.*, **3**, 176–181.

TANNOCK, I.F. & RATIN, P. (1989). Acid pH in tumours and its potential in therapeutic exploitation. *Cancer Res.*, **49**, 4373–4384.

VALERIOTE, F. & PUTTEN, L. (1975). Proliferation-dependent cytotoxic action of anti-cancer agents: a review. *Cancer Res.*, **35**, 2619–2630.

VINGERHOEDS, M.H., HAISMA, H.J., VAN MUIJEN, M., VAN DE RIJT, R.B.J., CROMMELIN, D.J.A. & STORM, G. (1993). A new application for liposomes in cancer therapy. *FEBS Lett.*, **336**, 485–490.

VITETTA, E.S., THORPE, P.E. & UHR, J.W. (1993). Immunotoxins: magic bullets or misguided missiles: *Trends Pharmacol. Sci.*, **14**, 148–154.

WALDMANN, T.A. (1991). Monoclonal antibodies in diagnosis and therapy. *Science*, **252**, 1657–1662.

WEINER, L.M., O'DWYER, J., KITSON, J., COMIS, R.L., FRANKEL, A.E., BAUER, R.J., KONRAD, M.S. & GROVES, E.S. (1989). Phase I evaluation of an anti-breast carcinoma monoclonal antibody 260F9-recombinant ricin A chain immunoconjugate. *Cancer Res.*, **49**, 4062–4067.

WORRALL, A.F. & CONNOLLY, B.A. (1990). The chemical synthesis of a gene codong for bovine pancreatic DNase I and its cloning and expression in *Escherichia coli*. *J. Biol. Chem.*, **265**, 21889–21895.

WU, Y., MIKULSKI, S.M., ARDELT, W., RYBAK, S.M. & YOULE, R.J. (1993). A cytotoxic ribonuclease. *J. Biol. Chem.*, **268**, 10686–10693.